# Supplementary figures and images for: Arabidopsis TRM5 encodes a nuclear-localised bifunctional tRNA guanine and inosine-N1-methyltransferase that is important for growth
Source: PLoS One. 2019 Nov 22;14(11):e0225064. doi: 10.1371/journal.pone.0225064 (PMC6874348; doi:10.1371/journal.pone.0225064)

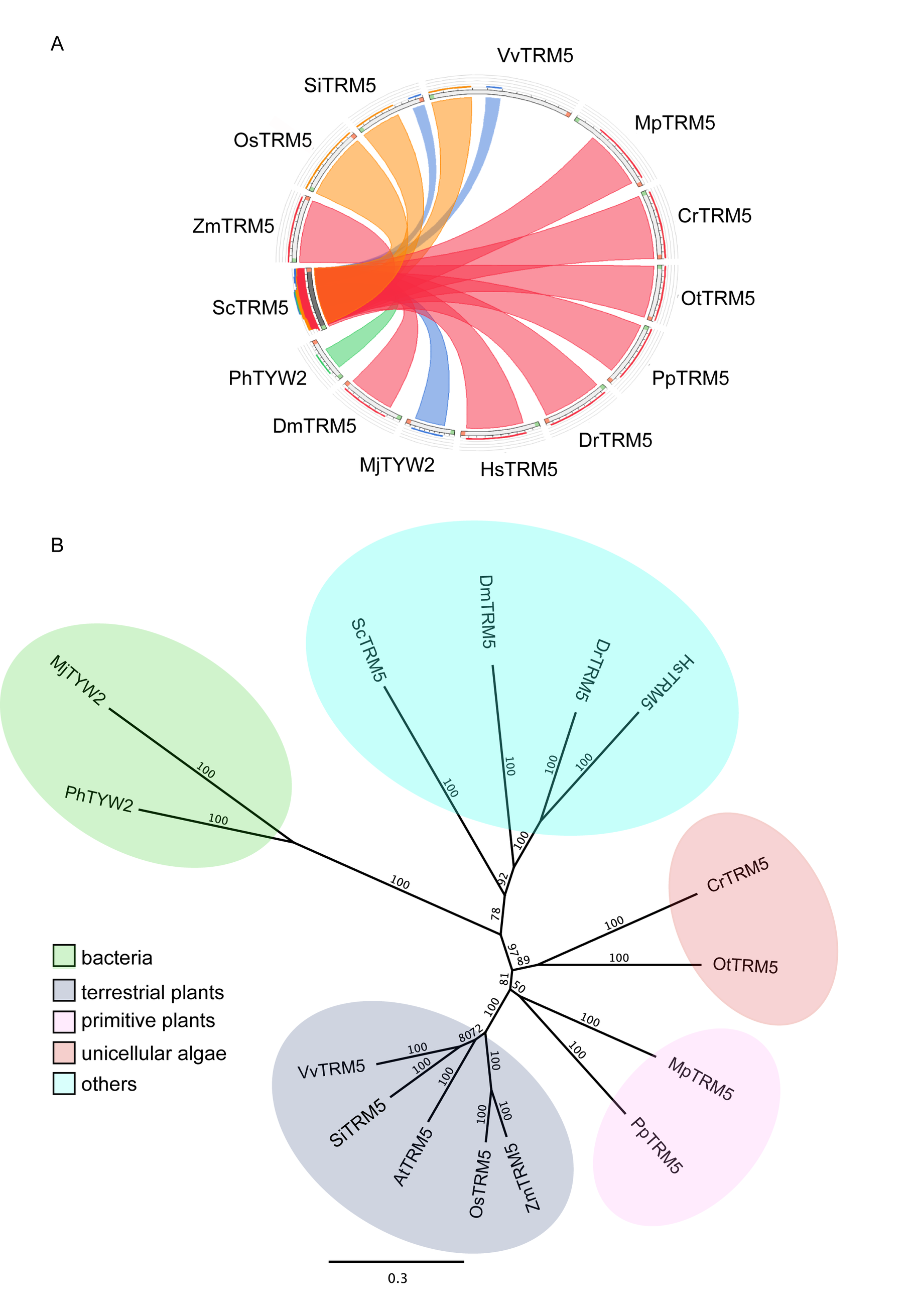

Supplement: S1 Fig — (A) Circos plot of sequence conservation of TRM5 orthologues in yeast (Sc), tomato (Sl), grape (Vv), Arabidopsis (At), maize (Zm), rice (Os), Marchantia (Mp), Physcomitrella (Pp), Chlamydomonas (Cr),Ostreococcus (Ot), humans (HsTrm5), Drosophila melanogaster (DmTrm5), Pyrococcus horikoshii (PhTYW2), and Methanococcus jannaschii (MjTYW2). The ribbons were coloured based on sequence identity, with blue < = 25%, green 25–50%, orange 51–75% and red for 76–99%. (B) Unrooted phylogenetic tree of the same TRM5 orthologues used to for sequence conservation analysis. (TIF) [file pone.0225064.s001.tif]

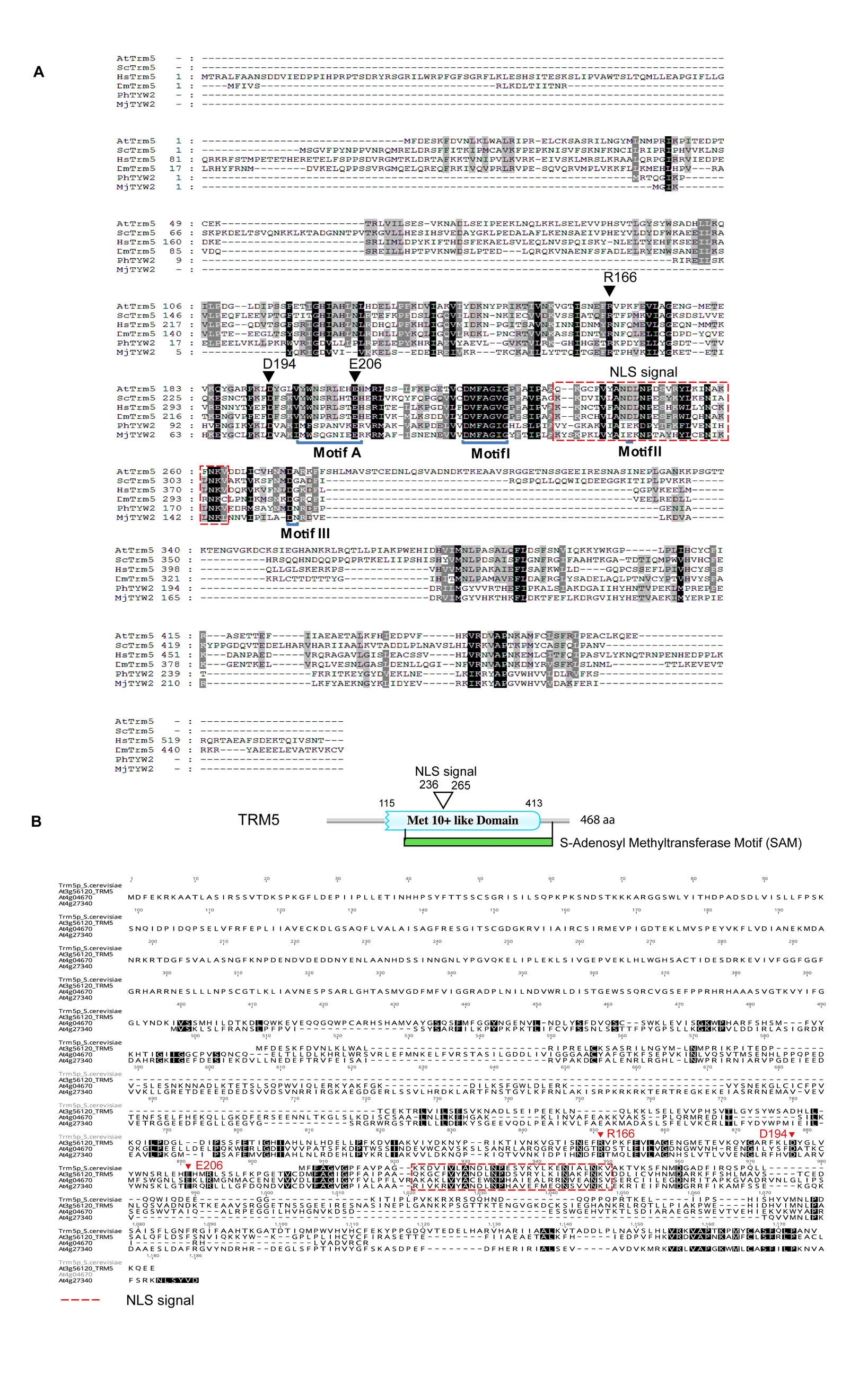

Supplement: S2 Fig — (A) Multiple sequence alignment of TRM5 proteins from Arabidopsis thaliana (At), yeast (ScTrm5), humans (HsTrm5), Drosophila melanogaster (DmTrm5), Pyrococcus horikoshii (PhTYW2), and Methanococcus jannaschii (MjTYW2). Black shaded boxes are identical across all species. Light shaded boxes are similar and nearly conserved residues. Asterisk indicates catalytic important amino acids. The predicted 29 aa importin α-dependent NLS is boxed in red. (B) Multiple sequence alignment of yeast Trm5p, Arabidopsis TRM5 (At3g56120) and the two closest related proteins from Arabidopsis. Black shaded boxes are conserved in at least 2 sequences. Met 10+ like domain and S-adenosyl Methyltransferase Motif (SAM) are detected in Arabidopsis TRM5 using NCBI Conserved Domains Search (https://www.ncbi.nlm.nih.gov/Structure/cdd/wrpsb.cgi). The position of importin α-dependent NLS is also indicated. (TIF) [file pone.0225064.s002.tif]

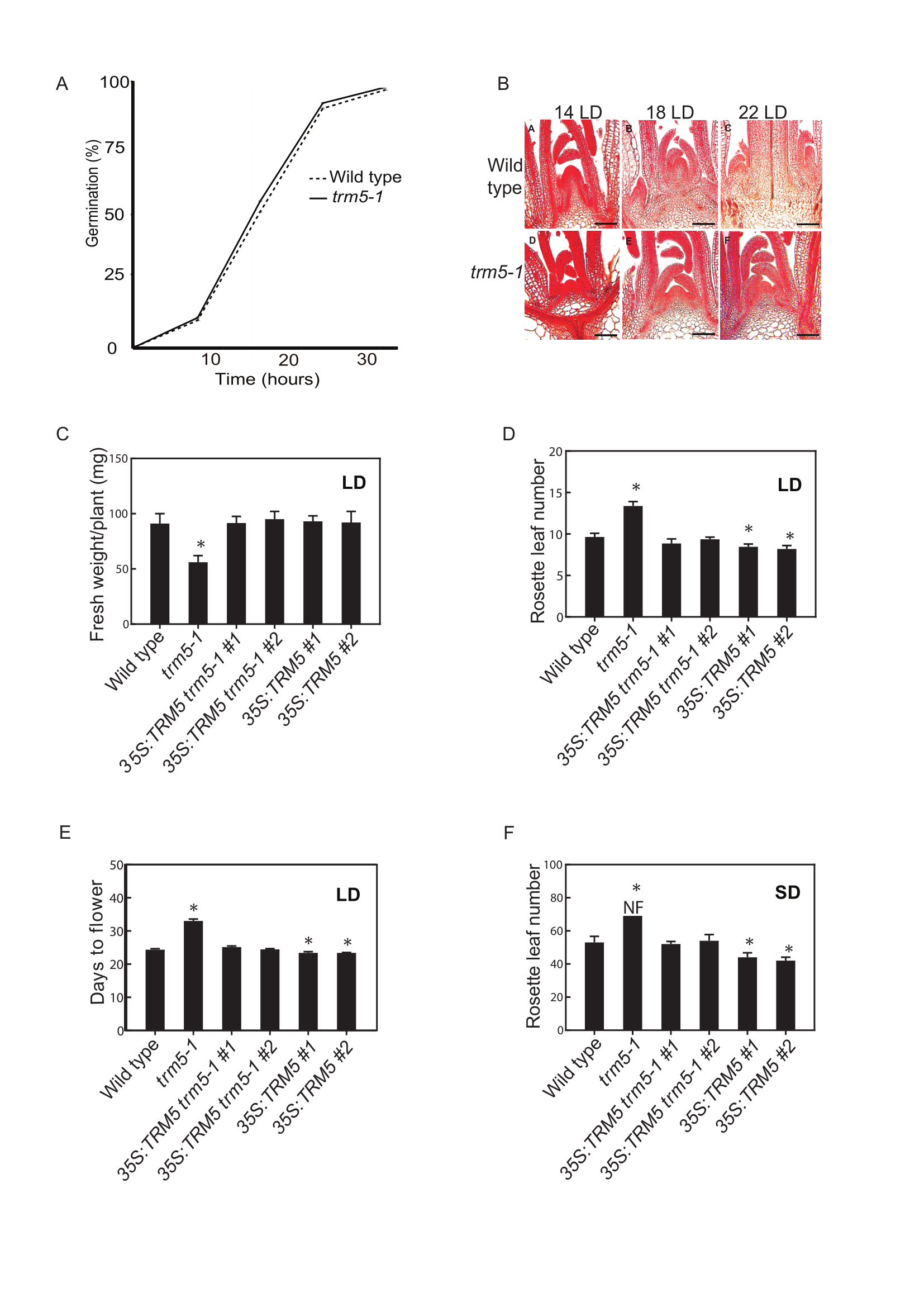

Supplement: S3 Fig — (A) Seeds (n = 100) of wild type and trm5-1 were sown on ½ MS plates, stratified at 4 oC and then grown at 21 oC under long day conditions for 32 hours. Germination was measured at 8, 16, 24 and 32 hours after shifting to 21 oC. (B) Sections of the shoot apical meristems of wild type and trm5-1 plants grown under long days for 14, 18 and 22 days. (C) The average fresh plant weight of long day grown plants. (D) The average rosette leaf number at flowering; (E) The average days to flowering under long days. (F) The rosette leaf number under short days. Data presented are means. Error bars are ± SE (n = 16). NF = did not flower. An asterisk indicates a statistical difference (P<0.05) as determined by Student’s t-test. (TIF) [file pone.0225064.s003.tif]

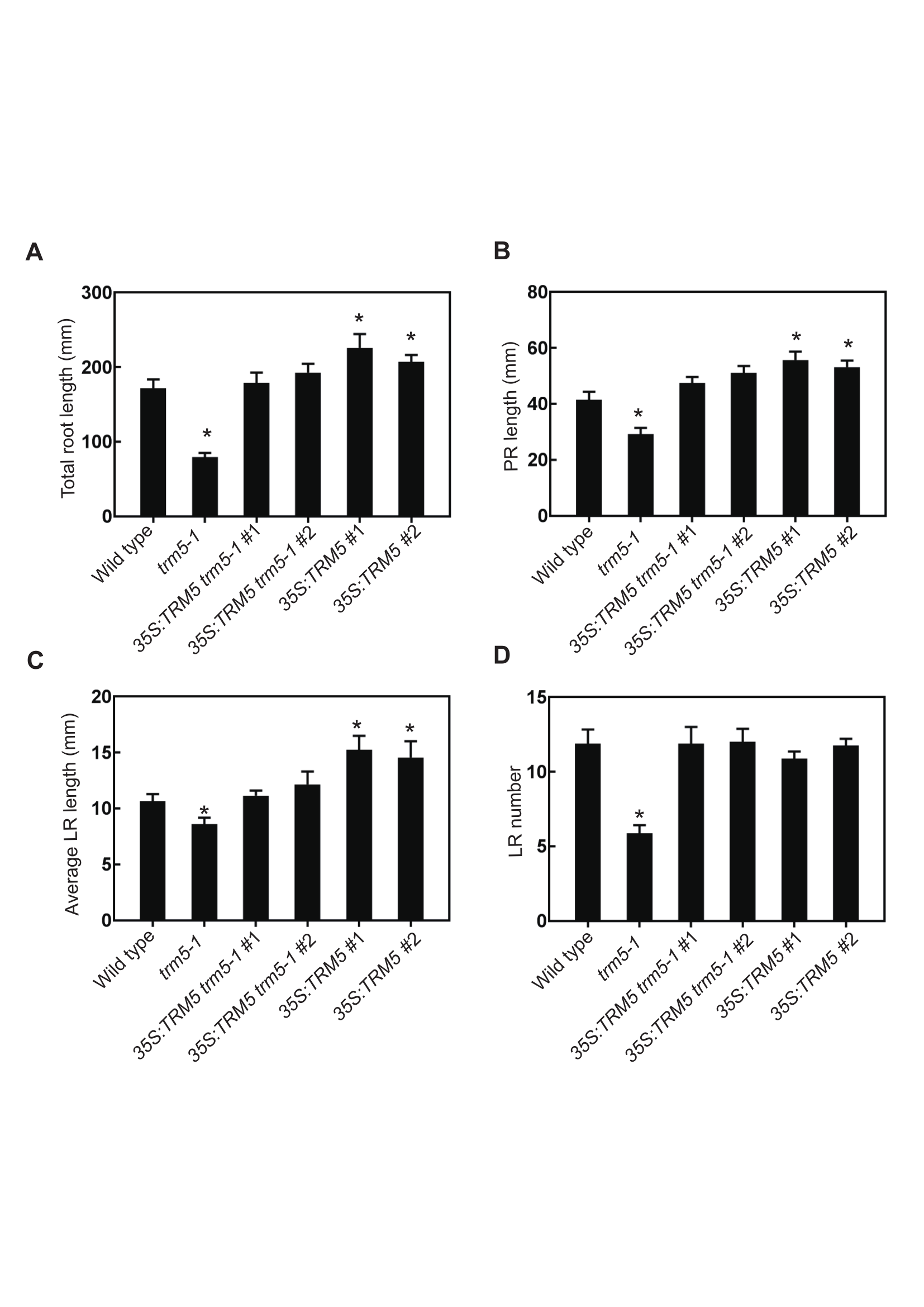

Supplement: S4 Fig — Seedlings of wild type, trm5, complemented lines (35S:TRM5 trm5-1), TRM5 overexpression lines (35S:TRM5) were vertically grown on ½ MS medium for 10 days and then measured. (A) Total root length, (B) Primary root, (PR) length, (C) average lateral root (LR) length and (D) LR number were measured 10 days after germination. Data presented are means. Error bars are ± SE (n = 10 plants). (TIF) [file pone.0225064.s004.tif]

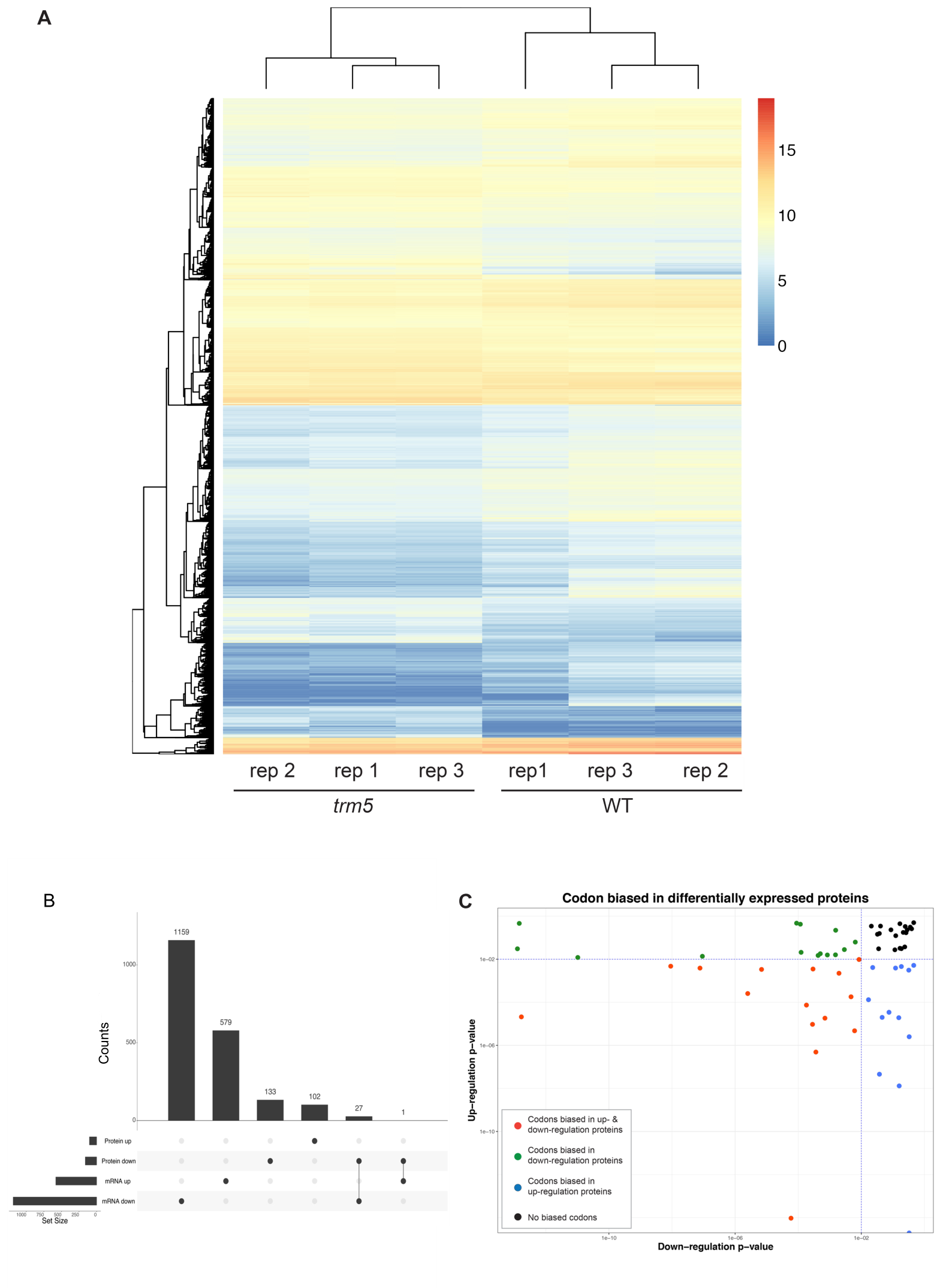

Supplement: S5 Fig — (A) RNA was purified from 10-day-old seedlings of wild type (wt) and trm5-1 (n = 3). RNA-seq analysis was performed and differentially abundant transcripts were hierarchically clustered. (B) An upset plot showing positive overlapping mRNAs and proteins identified by RNA-seq and proteomics analysis. (C) Codon bias analysis of codons in the up and down regulated proteins identified by proteomics analysis. (TIF) [file pone.0225064.s005.tif]
