# Supplementary material for: Arabidopsis TRM5 encodes a nuclear-localised bifunctional tRNA guanine and inosine-N1-methyltransferase that is important for growth
Source: PLoS One. 2019 Nov 22;14(11):e0225064. doi: 10.1371/journal.pone.0225064 (PMC6874348; doi:10.1371/journal.pone.0225064)
Supplement: S1 Table — (DOCX) [file pone.0225064.s006.docx]

**Supplementary Table S1.** Oligonucleotide primers used in this study.

| **RT PCR Primers** | **Sequence** |
| --- | --- |
| TRM5-qRTPCR_F | 5'-ACACGCAAATAAGAGATTGAGAC-3' |
| TRM5-qRTPCR_R | 5'-TGGAACTTCAAAGCAGTTTCAGC-3' |
| FT- qRTPCR_F | 5'-GCTACAACTGGAACAACCTTTGGC-3' |
| FT- qRTPCR_R | 5'-TGAATTCCTGCAGTGGGACTTGG-3' |
| CO- qRTPCR_F | 5'-CTACAACGACAATGGTTCCATTAAC-3' |
| CO- qRTPCR_R | 5'-CAGGGTCAGGTTGTTGC-3' |
| GI- qRTPCR_F | 5'-GGGTAAATATGCTGCTGGAGA-3' |
| GI- qRTPCR_R | 5'-CAGTATGACACCAGCTCCATT-3' |
| SOC1- qRTPCR_F | 5'-AGCTGCAGAAAACGAGAAGCTCTCTG-3' |
| SOC1-qR | 5'-GGGCTACTCTCTTCATCACCTCTTCC-3' |
| LFY- qRTPCR_F | 5'-ATCGCTTGTCGTCATGGCTG-3' |
| LFY- qRTPCR_R | 5'-GCAACCGCATTGTTCCGCTC-3' |
| AP1- qRTPCR_F | 5'-CAGCAGCACCAAATCCAGC-3' |
| AP1- qRTPCR_R | 5'-GAGCCTAGCCACTATTTATATG-3' |
|  |  |
|  |  |
|  |  |
|  |  |
| CCA1- qRTPCR_F | 5'-CCTCAA ACTTCA GAGTCCAATGC-3' |
| CCA1- qRTPCR_R | 5'-GACCCTCGTCAGACACAGACTTC-3' |
| LHY- qRTPCR_F | 5'-GAAGTCTCCGAAGAGGGTCG-3' |
| LHY- qRTPCR_R | 5'-TATTCACATTCTCTGCCACTTGAG-3' |
| TOC1- qRTPCR_F | 5'-GCTATGAACAGAAGTAAAGATTCG-3' |
| TOC1- qRTPCR_R | 5'-GGATATCCCGTCATTCCATTCGGA-3' |
| PRR7- qRTPCR_F | 5'-CTGCACTCGTTATATCGTTACTG-3' |
| PRR7- qRTPCR_R | 5'-GGCATGATCACCTCTGTTAG-3' |
| EF-1-α- qRTPCR_F | 5'-TGAGCACGCTCTTCTTGCTTTCA-3' |
| EF-1-α- qRTPCR_R | 5'-GGTGGTGGCATCCATCTTGTTACA-3' |
| **Primer sequences for T-DNA insertion identification** | |
| TRM5-1_022617RP | 5'-TTGCGAGGATAATTTGCAATC-3' |
| TRM5-1_022617LP | 5'-CCTTGTTTGGAGCAACATCTC-3' |
| TRM5-2_ 032376RP | 5’- CATGATGAGCTCCTTCCATTCAAAG-3’ |
| TRM5-2_ 032376LP | 5’- CGCATGTGCTCATGTTCCAATC-3’ |
| LB-SALK-b1.3 | 5'-ATTTTGCCGATTTCGGAAC-3' |
| **Primer sequences for *TRM5*** | |
| TRM5-CDSf1 | 5'-CACCATGTTTGATGAAAGCAAGTTCGATGTC-3' |
| TRM5-CDSr1 | 5'-CTCTTCTTGCTTCAAGCATGCTTC-3' |
| TRM5Pro_F1 | 5'-agtggctgatctgagggacggaagagg-3' |
| TRM53’_R1 | 5'-gagagctgcagaagacgccattggaag-3' |
| **Primer sequences for tRNA^Ala^ (AGC) amplification** | |
| tRNA-Ala-f | 5'-GGGGATGTAGCTCAGATGGTAG-3' |
| tRNA -Ala-f | 5'-TGGTGGAGATGCGGGGTATC-3' |
| **Probe sequences for tRNA^Ala^ (AGC) aminoacetylation assay** | |
| tRNA-Ala(AGC)-f | 5’-GGGGATGTAGCTCAGATGGTAG-3’ |
| tRNA-Ala(AGC)-R | 5’-TGGTGGAGATGCGGGGTATC-3’ |
| **Probe sequences for tRNA^Ala^ (AGC) and tRNA^Asp^ (GTC) northern blot analysis** | |
| tRNA^Asp(GTC)^ | 5’- GACAGGCGGGAATACTTACCACTATACTACAACGAC -3’/DIG_N |
| tRNA^Arg(CCT)^ | 5’- GCTAAGCGAGCGCTCTACCATCTGAGCTACATCCCC - 3’/DIG_N |
| tRNA^Ala(AGC)^ | 5’- AGGAAACAGACGCTCTATCCACTGAGCTACAGGCGC -3’/DIG_N |
