# Supplementary material for: Arabidopsis TRM5 encodes a nuclear-localised bifunctional tRNA guanine and inosine-N1-methyltransferase that is important for growth
Source: PLoS One. 2019 Nov 22;14(11):e0225064. doi: 10.1371/journal.pone.0225064 (PMC6874348; doi:10.1371/journal.pone.0225064)
Supplement: Attachment — Submitted filename: Rebuttal letter draft_2.0.docx [file pone.0225064.s009.docx]

**Please find below a point-by-point response to the editor, reviewers one and two comments.**

Reponses are in blue coloured font.

Editor’s comments:

(1) Comment- As the link between Figure S6 and the proteomic data remains somewhat unclear (see the reviewer's comments below), I would suggest removing this figure from the manuscript, provided that you clearly state elsewhere in the manuscript that the results have not been subjected to validation by an alternative method.

Response -The supplementary figure S6, along with the figure captions and all previous mentions in the manuscript have been removed. We have also added a senetence that in the future that validation of the proteomics data will be required via quantitative western blotting to verify the direct or indirect effect of the loss of TRM5. Please refer to page 14 lines 324-327 of the manuscript for the edits.

(2) Comment- I am also asking you to supply additional information on some aspects of Supplementary Table S3, Please clarify how the p-values in the table have been adjusted (i.e. corrected for multiple testing), as this information is not included in the Methods section. Please note that if they have not been corrected, the validity of some of your conclusions might be compromised. In this table, please also include the raw values for each genotype (i.e. not only their ratios).

Response - Additional information on the proteomics data, including the raw values of each genotype have been included in the updated Supplementary Table 3. The p-values have been corrected using Benjamini-Hochberg (BH) correction method and the methods section in the manuscript has been updated accordingly. The BH corrected p-values have been included in supplementary Table 3.

Additional Editor Comments (if provided):

(3) Comment- Line 47: Split the two sentences "...mutants. However, ..."

Response - The suggested edits were incorporated. The sentences are split into two at page 2 line 47-48.

(4) Comment- l. 85: remove comma before "catalyses"

Response -The sentence has been amended with the comma removed. Please refer to page 4 line 86.

(5) Comment- l. 89: add a comma after "TrmD".

Response - A comma has been added after TrmD. Please refer to page 4 line 89.

(6) Comment- l. 91: have -> has

Response - This sentence was corrected as suggested. Please refer to page 4 line 91.

(7) Comment- Line 150-151: "in Arabidopsis thaliana" is said twice

Response - The redundant “in *Arabidopsis thaliana*” mentioned in the manuscript was removed as suggested. Please refer to page 6 line 151.

(8) Comment- Line 186: Use uppercase characters for the gene name. Regulators -> REGULATOR (no S)

Response - The gene name *PSEUDO RESPONSE REGULATOR 7* has been corrected to all upper-case characters at page 8 line 190.

(9) Comment- Line 198: something is wrong in this sentence ("a yeast")

Response - The sentence was amended by removing “..a yeast”. Please refer to page 8 line 201.

(10) We proofread our manuscript prior to resubmission.

With this resubmission, we attached the corrected Fig 1, which in Fig 1 panel A and B the misspelled “SITRM5” has been corrected to “SiTRM5”.

Reviewer #1:

We thank reviewer 1 for the positive feedback on the manuscript revisions.

Reviewer #2:

(11) Now that western validation data are available, the interpretation that the results were consistent with the proteomics needs to be supported with much more analysis. I see no names of proteins identified in Figure 7 (call the dots out on the proteomics that were tested in the western, as is commonly done) or discussed in the text so that I can evaluate how the western data do, or do not, support the proteomics by a direct comparison.

     - major issue: there is no quantification provided in Figure S6- I see 6 proteins, with some whose levels seem to change to some extent relative to WT and some that don't appear to change as much, but of course this would require actual quantification in some way. The problem is normalization for loading, which the authors suggest is supposed to be done visually by the coomassie staining, although this is of course not appropriate at all because we are looking at one protein (which one?) and it is not the typical way to normalize by a protein western control signal for a protein that should not be changing. However, even trying to interpret this result (by eye) seems to clearly show heavier bands in the coomassie WT "100%" lane than in the corresponding trm5-1 lane. As presented, these data definitely do not meet typical standards for scientific rigor.

     - were some of these proteins controls that are used to show that levels did not change, as expected based on the proteomics?

     - I assume the 100% listed for trm5-1 indicates some fraction of the sample loaded, but this doesn't quite make sense- this is better phrased with some indication of exact total protein loaded per lane? This is a minor point, but would help improve clarity.

     Bottom line, the superficial way the validation is performed and described significantly impacts the confidence in the proteomics data, which are a major conclusion of the paper.

Response - The supplementary figure S6 has been removed from the manuscript after careful revision of this manuscript based on the reviewer #2’s comments.

(12) a related issue is with the RNAseq data used to suggest targets whose regulation is at the level of translation. Since validation of the RNAseq data are not provided (which is the standard in the field), and because now it is clear that the western validation (now shown) did not focus specifically on any of these proteins, and the quantitative data needed to rigorosuly support the validity of the proteomics are also absent (see point above), this result identifying some proteins whose may be regulated at the level of translation can not be viewed as well-supported by any current standards in the field. This highly speculative result could be removed to discussion, or quite honestly from the paper entirely, without further substantiation.

Response - We have edited the manuscript and have soften our results section according to the reviewer’s suggestion. Please refer to page 14 lines 324-327 of the manuscript for the edits.

We thank reviewer 2 for the constructive feedback on the manuscript.
